# Diagnosing Discogenic Low Back Pain Associated with Degenerative Disc Disease Using a Medical Interview

**DOI:** 10.1371/journal.pone.0166031

**Published:** 2016-11-07

**Authors:** Juichi Tonosu, Hirohiko Inanami, Hiroyuki Oka, Junji Katsuhira, Yuichi Takano, Hisashi Koga, Yohei Yuzawa, Ryutaro Shiboi, Yasushi Oshima, Satoshi Baba, Sakae Tanaka, Ko Matsudaira

**Affiliations:** 1 Department of Orthopedic Surgery, Kanto Rosai Hospital, Kanagawa, Japan; 2 Department of Orthopedic Surgery, Inanami Spine and Joint Hospital, Tokyo, Japan; 3 Department of Orthopedic Surgery, Iwai Orthopaedic Medical Hospital, Tokyo, Japan; 4 Department of Medical Research and Management for Musculoskeletal Pain, 22nd Century Medical and Research Center, Faculty of Medicine, The University of Tokyo, Tokyo, Japan; 5 Department of Orthopedic Surgery, Faculty of Medicine, The University of Tokyo, Tokyo, Japan; 6 Department of Prosthetics & Orthotics and Assistive Technology, Faculty of Medical Technology, Niigata University of Health and Welfare, Niigata, Japan; Northwestern University Feinberg School of Medicine, UNITED STATES

## Abstract

**Purposes:**

To evaluate the usefulness of our original five questions in a medical interview for diagnosing discogenic low back pain (LBP), and to establish a support tool for diagnosing discogenic LBP.

**Materials and Methods:**

The degenerative disc disease (DDD) group (n = 42) comprised patients diagnosed with discogenic LBP associated with DDD, on the basis of magnetic resonance imaging findings and response to analgesic discography (discoblock). The control group (n = 30) comprised patients with LBP due to a reason other than DDD. We selected patients from those who had been diagnosed with lumbar spinal stenosis and had undergone decompression surgery without fusion. Of them, those whose postoperative LBP was significantly decreased were included in the control group. We asked patients in both groups whether they experienced LBP after sitting too long, while standing after sitting too long, squirming in a chair after sitting too long, while washing one’s face, and in the standing position with flexion. We analyzed the usefulness of our five questions for diagnosing discogenic LBP, and performed receiver operating characteristic (ROC) curve analysis to develop a diagnostic support tool.

**Results:**

There were no significant differences in baseline characteristics, except age, between the groups. There were significant differences between the groups for all five questions. In the age-adjusted analyses, the odds ratios of LBP after sitting too long, while standing after sitting too long, squirming in a chair after sitting too long, while washing one’s face, and in standing position with flexion were 10.5, 8.5, 4.0, 10.8, and 11.8, respectively. The integer scores were 11, 9, 4, 11, and 12, respectively, and the sum of the points of the five scores ranged from 0 to 47. Results of the ROC analysis were as follows: cut-off value, 31 points; area under the curve, 0.92302; sensitivity, 100%; and specificity, 71.4%.

**Conclusions:**

All five questions were useful for diagnosing discogenic LBP. We established the scoring system as a support tool for diagnosing discogenic LBP.

## Introduction

Low back pain (LBP) affects most adults at some point in their lives. In the last decade, LBP was continuously found to be the top leading cause of years lived with disability globally [1]. As in many industrialized countries, LBP is one of the most common health disabilities in Japan. In a population-based survey, the lifetime and 4-wk LBP prevalence were 83% and 36%, respectively [2].

It has been difficult to identify the cause of LBP. A specific source of pain can be identified in some cases of LBP; however, the source cannot be identified in other cases of LBP (i.e., non-specific LBP) [3]. Magnetic resonance imaging (MRI) can identify underlying pathologies of LBP. However, the importance of MRI findings is unclear and controversial. Some reports have shown that disc degeneration was a source of LBP [4,5], whereas other reports have shown that there was no relationship between disc degeneration and LBP [6,7]. Reports have also shown that discogenic LBP associated with degenerative disc disease (DDD) is confirmed by the MRI findings and response to the injection of contrast media or local anesthesia into the disc [8–10]. Schwarzer et al. reported that 39% of cases of chronic LBP are discogenic, and the diagnosis is made by computed tomography after discography [11]. The technique of injecting local anesthesia into a disc is analgesic discography (discoblock). However, these procedures do not necessarily indicate high specificity findings of discogenic LBP [12, 13], and they are invasive and harmful to the disc [14, 15].

We hypothesized that discogenic LBP is one of the causes of LBP, and we sought to determine easier and less invasive means of diagnosing discogenic LBP. Few reports have specified that LBP in the sitting position can indicate discogenic LBP [16]. However, no report has found that LBP in standing position with flexion can indicate discogenic LBP. Based on our clinical experiences, we also hypothesized that discogenic LBP could be indicated in standing position with flexion and in sitting position. The purpose of the current study was to evaluate the usefulness of our original questions in a medical interview about LBP, which was intended to determine the characteristics of discogenic LBP, and establish a support tool for diagnosing discogenic LBP.

## Materials and Methods

### Subjects

**Fig 1:**
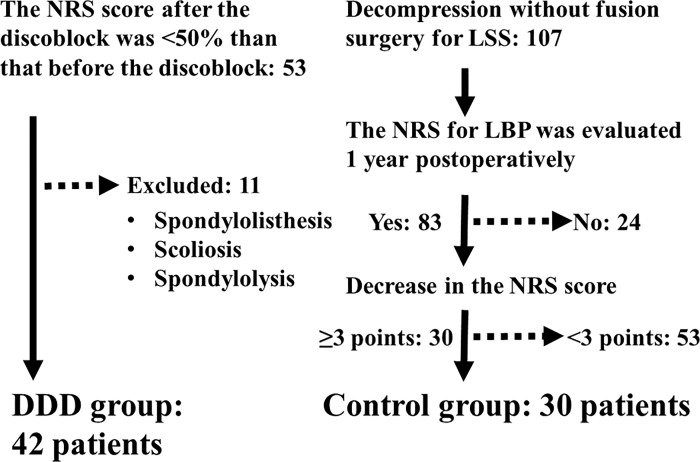
Study flow chart. Forty-two patients were included in the degenerative disc disease (DDD) group, and 30 patients were included in the control group. LBP, low back pain; NRS, numerical rating scale; LSS, lumbar spinal stenosis.

In the current study, we defined the DDD group as those who suffered from discogenic LBP associated with DDD. The DDD group consisted of consecutive patients from November 2012 to April 2014. Fifty-three patients had been diagnosed as having DDD by MRI and discoblock. Of 53 patients, we excluded 11 who had suffered from spondylolisthesis, scoliosis, and spondylolysis accompanied by DDD. Therefore, the DDD group consisted of 42 consecutive patients. We defined the control group as those who had suffered from LBP due to reasons other than DDD. We selected the control group from patients who had been diagnosed as having lumbar spinal stenosis (LSS) and had undergone posterior decompression surgery without fusion. The control group consisted of consecutive patients from April 2012 to December 2013. One hundred and seven patients had undergone decompression surgery for LSS. Of 107 patients, we could evaluate the numerical rating scale (NRS) score of 83 patients’ LBP at 1 year postoperatively. Of 83 patients, 30 had a decrease in the postoperative NRS score of greater than or equal to 3 points compared with the preoperative NRS score. We included these 30 patients in the control group. In summary, 72 patients were included in this study, which consisted of 42 in the DDD group and 30 in the control group (Fig 1). We also collected patients’ background information, including their age, sex, height, weight, and smoking habit, using a self-written questionnaire. We calculated the body mass index (BMI) from the data of height and weight. We also determined the NRS score of patients’ LBP and assessed the Oswestry Disability Index (ODI) score [17] using a self-written questionnaire. We used a validated version of the Japanese ODI, which had been translated from the ODI version 2.0 [18]. The reliability and validity of this version was evaluated in their previous study, and was sufficient to use for outcome studies in Japan. This study was approved by the medical/ethics review board of Iwai Orthopaedic Medical Hospital. Written informed consent was obtained from all the patients.

### Definition of discogenic LBP

Although there is no consensus on how to diagnose discogenic LBP, we hypothesized and defined discogenic LBP as LBP that met the following criteria: MRI findings of a degenerated disc, and response to the discoblock into the disc suggestive of LBP. Although a discoblock may be harmful to the disc and it does not necessarily indicate high specificity findings of discogenic LBP, we hypothesized that a positive response to a discoblock indicates discogenic LBP.

At our hospital, well-trained medical clerks ask all patients about their medical history and symptoms during their first visit usually before they see the doctor. For patients who had suffered from lumbar diseases, medical clerks asked them about the following items in a medical interview: whether they had LBP from sitting too long, whether they had LBP while standing after sitting too long, whether they squirmed in a chair after sitting too long, whether they had LBP while washing their face, and whether they had LBP in standing position with flexion via a medical interview. Additionally, we precisely defined and evaluated the region of LBP and depicted it in a diagram for patients (i.e., pain localized between the costal margin and the inferior gluteal folds according to a previous report [19,20]). This was important for standardizing the study protocol for LBP.

We evaluated patients’ physical findings, and radiography and MRI findings as needed. If the MRI showed only disc degeneration without disc herniation, spinal stenosis, or any other obvious findings, we suspected DDD. We evaluated disc degeneration on sagittal T2-weighted MRI based on Pfirrmann’s grading system [21]. We considered grades ≥4 as disc degeneration. When we suspected discogenic LBP associated with DDD and the patient’s disability was severe despite conservative therapies, we performed an additional examination (discoblock, a 1-mL injection of 1% lidocaine into the disc suggestive of LBP), and evaluated the degree of LBP both before and after the injection. We hypothesized discogenic LBP associated with DDD when the NRS score for LBP after the discoblock was <50% of that before the discoblock, although it was unclear whether the cutoff reduction rate of 50% was appropriate. When multilevel disc degeneration was shown on MRI, we performed the injections on a different day and evaluated the effectiveness of the injection for each disc.

### Statistical methods

We compared the baseline characteristics of both groups, and analyzed the usefulness of the aforementioned five items of the medical interview for diagnosing discogenic LBP. For the age-adjusted analysis, we set the cut-off value at 65 years. Moreover, after identifying significant symptoms of discogenic LBP, we developed a support tool for diagnosing discogenic LBP.

Descriptive statistics were determined and presented as means and standard deviations or frequencies and percentages. Between-group differences in baseline characteristics were evaluated using the chi-square test for categorical variables, and Student’s t-test for continuous variables. Age-adjusted odds ratios and 95% confidential intervals for each questionnaire were evaluated by logistic regression analyses. Moreover, we set the scores of each item as integral values from each age-adjusted odds ratio, and performed receiver operating characteristic (ROC) curve analysis to develop a support tool for diagnosing discogenic LBP. Finally, we calculated the area under the ROC curve (AUC), sensitivity, and specificity. An AUC of 1.0 indicated perfect discrimination, and in general, an AUC ≥0.7 was considered to indicate acceptable discrimination. Statistical analysis was performed using the JMP 11.0 software program (SAS Institute, Cary, NC, USA). A p value <0.05 was considered significant.

## Results

Patients’ average age was 53.9 years in the DDD group and 71.1 years in the control group. The ratio of age ≥65 years was 16.7% in the DDD group and 30.1% in the control group. There was a significant difference in age between the DDD and control groups (p < 0.0001 and p = 0.0002, respectively). However, there were no significant differences in the other baseline characteristics such as sex, BMI, smoking habit, NRS score, and ODI score. There were significant differences between the groups for each item of the medical interview about LBP after sitting too long (p < 0.0001), LBP while standing after sitting too long (p < 0.0001), squirming in a chair after sitting too long (p = 0.011), LBP while washing one’s face (p < 0.0001), and LBP in standing position with flexion (p < 0.0001) (Table 1).

**Table 1:** Baseline characteristics of patients in the DDD group and control group.

	DDD group (n = 42)	Control group (n = 30)	*P* value
● Age^1)^	53.4 ± 16.2	71.1 ± 9.4	<0.0001*
● Age ≥65 years^2)^	12 (16.7)	22 (30.1)	0.0002*
● Female sex^2)^	15 (35.7)	7 (23.3)	0.26
● BMI (kg/m^2^)^1)^	24.2 ± 3.2	24.9 ± 2.7	0.36
● Smoking habit^2)^	28 (66.7)	16 (53.3)	0.25
● Current smoking habit^2)^	9 (21.4)	6 (20.0)	0.88
● NRS score^1)^	6.2 ± 2.3	6.2 ± 1.7	0.99
● ODI score^1)^	37.2 ± 13.3	37.8 ± 9.9	0.84
● LBP after sitting too long^2)^	35 (83.3)	9 (30.0)	<0.0001*
● LBP while standing after sitting too long^2)^	35 (83.3)	11 (36.7)	<0.0001*
● Squirming in a chair after sitting too long^2)^	33 (78.6)	15 (50.0)	0.011*
● LBP while washing one’s face^2)^	31 (73.8)	6 (20.0)	<0.0001*
● LBP in standing position with flexion^2)^	22 (52.4)	2 (6.7)	<0.0001*

Data are shown as mean ± standard deviation or number of participants (%).

*: *P* < 0.05

^1)^: Student’s t-test

^2)^: chi-square test.

DDD, degenerative disc disease; ODI, Oswestry Disability Index; NRS, numerical rating scale; LBP, low back pain.

In the age-adjusted analyses, the odds ratios of LBP after sitting too long, LBP while standing after sitting too long, squirming in a chair after sitting too long, LBP while washing one’s face, and LBP in standing position with flexion were 10.5, 8.5, 4.0, 10.8, and 11.8, respectively. There were significant differences for all five items of the medical interview between the groups (Table 2). The integer scores were 11, 9, 4, 11, and 12, respectively, and the sum of the points of the five scores ranged from 0 to 47. Results of the ROC analysis were as follows: cut-off value, 31 points; AUC, 0.92302; sensitivity, 100%; and specificity, 71.4%.

**Table 2:** Age-adjusted odds ratio, 95% confidential interval, and p-value for each item of the medical interview regarding LBP.

	Odds ratio	95% confidential interval	*P* value	Integer score
● LBP after sitting too long	10.5	3.3–39.4	<0.0001*	11
● LBP while standing after sitting too long	8.5	2.7–31.9	0.0002*	9
● Squirming in a chair after sitting too long	4.0	1.3–13.7	0.016*	4
● LBP while washing one’s face	10.8	3.3–41.3	<0.0001*	11
● LBP in standing position with flexion	11.8	2.8–82.2	0.0004*	12

*: *P* < 0.05.

LBP, low back pain.

## Discussion

We examined five items of our medical interview regarding discogenic LBP. We hypothesized and defined discogenic LBP as a degenerated disc on MRI and response to a discoblock used for the disc suggestive of LBP.

No significant difference was observed in the baseline characteristics between the two groups, except with regard to age. The DDD group consisted of significantly younger patients and thus had a wider generation than the control group, although disc degeneration progresses with advancing age [5]. The reason for this may be caused by our definition of the control group.

We intended to define the control group as those who had LBP that was not mild for reasons other than discogenic LBP. The NRS score of 6.2 and ODI score of 37.8 in the control group indicate that the LBP was not mild, thus it was equivalent to that in the DDD group in terms of severity. However, it is difficult to confirm whether LBP was discogenic. Accordingly, we focused on patients who had been diagnosed as having LSS. Some patients with LSS have LBP, whereas other patients do not have LBP. The MRI scans of patients with LSS often show degenerated discs in addition to spinal stenosis, because disc degeneration progresses with advancing age [5] and LSS is often present in older people. However, the cause of LBP in patients with LSS is not necessarily derived from disc degeneration. It can often improve after decompression surgery without fusion [22], which indicates that compression of the dura itself can present as LBP in these patients. We hypothesized that improvement of LBP in the control group resulted from decompression of the dura itself and that it was not associated with disc degeneration, although the lack of a negative response to the discoblock in the control group was not evaluated in this study. We excluded patients who had undergone decompression combined with fusion surgery because the improvement of LBP by fusion surgery implies the co-existence of discogenic LBP. We defined a clinically meaningful improvement in LBP postoperatively as a reduction in the NRS score of ≥3 points, according to a study that reported that the cut-off value for the decrease in the NRS score is 2.5 for successful lumbar surgery [23]. Therefore, we considered the cause of LBP in the control group as LBP caused by LSS itself and that it was not associated with DDD. As LSS is often present in more aged people, there was a significant difference in age between the two groups. Therefore, we performed age-adjusted analyses. We defined the cut-off age as 65 years. Since the prevalence of LBP increases with advancing age [2], we did not underestimate the DDD group, which was younger than the control group.

There were significant differences between the groups in all the five items of our medical interview for analyses adjusted and not adjusted by age. The odds ratios of only the items of LBP after sitting too long, LBP while washing one’s face, and LBP in standing position with flexion were >10. One reason for this may be because the results seemed to be associated with a higher intradiscal pressure in sitting and standing positions with flexion [24–28]. LBP while standing after sitting too long was significantly associated with discogenic LBP. The motion of standing after sitting too long includes changing the status of both the disc and facet, which is often degenerated in patients with LSS; however, our result indicated that LBP was discogenic. Therefore, the result may have been derived from the increasing intradiscal pressure. The items LBP while washing one’s face and LBP in standing position with flexion may be similar; however, we had intended to differentiate mild flexion of the trunk, such as the posture for washing one’s face from full flexion of the trunk. In terms of the results, both items can significantly indicate discogenic LBP. The results may have also been derived from increasing intradiscal pressure.

Another reason why the results seemed to be similar to previous reports may be because being in one position too long advances disc degeneration [29, 30]. We assumed that being in one position too long caused discogenic LBP. LBP after sitting too long was also significantly associated with discogenic LBP. The high odds ratio of 10.5 for sitting too long in the current study may have been derived from being in the same position too long rather than from increasing intradiscal pressure itself. The symptom of squirming in a chair after sitting too long was also significantly associated with discogenic LBP. There has been no report about the relationship between LBP and squirming in a chair after sitting too long. This may have also been derived from being in the same position too long.

The AUC of 0.92302 was considered to indicate acceptable discrimination. ROC analysis indicated the cut-off value of 31 in our scoring system for diagnosing discogenic LBP, which meant that >31 points of the total 47 points are needed to diagnose discogenic LBP. Considering each item of the medical interview, we can diagnose discogenic LBP in all cases if four or five of the five items are positive, and in some cases if three of five items are positive.

There were some limitations to the current study. First, answers to the medical interview were not necessarily accurate. The subjective evaluation of the patients’ own LBP can vary, i.e., positive or negative responses can differ depending on the medical interview. However, the results of the current study showed the high odds ratios of the five items, so we considered the results acceptable. Second, we did not evaluate the effectiveness of a discoblock in patients in the control group. The MRI scans of patients with LSS often show degenerated discs in addition to spinal stenosis. We omitted the evaluation of degenerated disc by discoblock in the control group. Patients had been diagnosed as having LSS based on clinical features such as lower limb symptoms and MRI findings; thus, an additional discoblock seemed unnecessary from clinical and ethical standpoints. However, if there were negative findings among the control group, our method for diagnosing discogenic LBP is more reliable. Third, we could not diagnose the degenerated disc responsible for LBP by the medical interview without performing the discoblock if there were multiple degenerated discs on the patient’s MRI. Fourth, we did not evaluate any other diseases such as LBP associated with sacroiliac joint dysfunction, LBP of the zygapophyseal joint, and major disturbances of the central nervous system associated with chronic pain. Fifth, there was selection bias among our patients. All patients in the DDD group had undergone surgery, although the therapy for discogenic LBP associated with DDD was usually considered conservative. Although patients in the DDD group who were sent to our hospital had a tendency of having severe LBP, patients from one hospital cannot represent patients with discogenic LBP in general.

## Conclusions

In accordance with our hypotheses that discogenic LBP exists and that a positive response to a discoblock indicates discogenic LBP, all five items of our medical interview about LBP (i.e., LBP after sitting too long, LBP while standing after sitting too long, squirming in a chair after sitting too long, LBP while washing one’s face, and LBP in standing position with flexion) were useful for diagnosing discogenic LBP associated with DDD. We can diagnose discogenic LBP in all cases if four or five of the five items of the medical interview are positive, and in some cases, if three of five items are positive.

## Supporting Information

S1 File Supporting information. Dataset of this study.
(XLS)

## References

[1] Vos T, Flaxman AD, Naghavi M, Lozano R, Michaud C, Ezzati M, et al Years lived with disability (YLDs) for 1160 sequelae of 289 diseases and injuries 1990–2010: a systematic analysis for the Global Burden of Disease Study 2010. Lancet. 2012;380: 2163–2196. 10.1016/S0140-6736(12)61729-2 23245607 PMC6350784

[2] Fujii T, Matsudaira K. Prevalence of low back pain and factors associated with chronic disabling back pain in Japan. Eur Spine J. 2013;22: 432–438. 10.1007/s00586-012-2439-0 22868456 PMC3555622

[3] Deyo RA, Weinstein JN. Low back pain. N Engl J Med. 2001;344: 363–370. 10.1056/NEJM200102013440508 11172169

[4] Kjaer P, Leboeuf-Yde C, Korsholm L, Sorensen JS, Bendix T. Magnetic resonance imaging and low back pain in adults: a diagnostic imaging study of 40-year-old men and women. Spine. 2005;30: 1173–1180. 15897832 10.1097/01.brs.0000162396.97739.76

[5] Cheung KM, Karppinen J, Chan D, Ho DW, Song YQ, Sham P, et al Prevalence and pattern of lumbar magnetic resonance imaging changes in a population study of one thousand forty-three individuals. Spine. 2009;34: 934–940. 10.1097/BRS.0b013e3181a01b3f 19532001

[6] Berg L, Hellum C, Gjertsen Ø, Neckelmann G, Johnsen LG, Storheim K, et al Do more MRI findings imply worse disability or more intense low back pain? A cross-sectional study of candidates for lumbar disc prosthesis. Skeletal Radiol. 2013;42: 1593–1602. 10.1007/s00256-013-1700-x 23982421

[7] Endean A, Palmer KT, Coggon D. Potential of magnetic resonance imaging findings to refine case definition for mechanical low back pain in epidemiological studies: a systematic review. Spine. 2011;36: 160–169. 10.1097/BRS.0b013e3181cd9adb 20739918 PMC3088902

[8] Eck JC, Sharan A, Resnick DK, Watters WC 3rd, Ghogawala Z, Dailey AT, et al Guideline update for the performance of fusion procedures for degenerative disease of the lumbar spine. Part 6: discography for patient selection. J Neurosurg Spine. 2014;21: 37–41. 10.3171/2014.4.SPINE14269 24980583

[9] Manchikanti L, Benyamin RM, Singh V, Falco FJ, Hameed H, Derby R, et al An update of the systematic appraisal of the accuracy and utility of lumbar discography in chronic low back pain. Pain Physician. 2013;16: SE55–SE95. 23615887

[10] Ohtori S, Kinoshita T, Yamashita M, Inoue G, Yamauchi K, Koshi T, et al Results of surgery for discogenic low back pain: a randomized study using discography versus discoblock for diagnosis. Spine. 2009;34: 1345–1348. 10.1097/BRS.0b013e3181a401bf 19440168

[11] Schwarzer AC, Aprill CN, Derby R, Fortin J, Kine G, Bogduk N. The prevalence and clinical features of internal disc disruption in patients with chronic low back pain. Spine. 1995;20: 1878–1883. 8560335 10.1097/00007632-199509000-00007

[12] Carragee EJ, Tanner CM, Khurana S, Hayward C, Welsh J, Date E, et al The rates of false-positive lumbar discography in select patients without low back symptoms. Spine. 2000;25: 1373–1380. 10828919 10.1097/00007632-200006010-00009

[13] Carragee EJ, Lincoln T, Parmar VS, Alamin T. A gold standard evaluation of the “discogenic pain” diagnosis as determined by provocative discography. Spine. 2006;31: 2115–2123. 10.1097/01.brs.0000231436.30262.dd 16915099

[14] Carragee EJ, Don AS, Hurwitz EL, Cuellar JM, Carrino JA, Herzog R. 2009 ISSLS Prize Winner: Does discography cause accelerated progression of degeneration changes in the lumbar disc: a ten-year matched cohort study. Spine. 2009;34: 2338–2345. 10.1097/BRS.0b013e3181ab5432 19755936

[15] Cuellar JM, Stauff MP, Herzog RJ, Carrino JA, Baker GA, Carragee EJ. Does provocative discography cause clinically important injury to the lumbar intervertebral disc? A 10-year matched cohort study. Spine J. 2016;16: 273–280. 10.1016/j.spinee.2015.06.051 26133255

[16] Young S, Aprill C, Laslett M. Correlation of clinical examination characteristics with three sources of chronic low back pain. Spine J. 2003;3: 460–465. 14609690 10.1016/s1529-9430(03)00151-7

[17] Fairbank JC, Couper J, Davies JB, O’Brien JP. The Oswestry low back pain disability questionnaire. Physiotherapy. 1980;66: 271–273. 6450426

[18] Fujiwara A, Kobayashi N, Saiki K, Kitagawa T, Tamai K, Saotome K. Association of the Japanese Orthopaedic Association score with the Oswestry Disability Index, Roland-Morris Disability Questionnaire, and short-form 36. Spine. 2003;28: 1601–1607. 12865852

[19] Krismer M, van Tulder M; Low Back Pain Group of the Bone and Joint Health Strategies for Europe Project. Strategies for prevention and management of musculoskeletal conditions. Low back pain (non-specific). Best Pract Res Clin Rheumatol. 2007;21: 77–91. 10.1016/j.berh.2006.08.004 17350545

[20] Dionne CE, Dunn KM, Croft PR, Nachemson AL, Buchbinder R, Walker BF, et al A consensus approach toward the standardization of back pain definitions for use in prevalence studies. Spine. 2008;33: 95–103. 10.1097/BRS.0b013e31815e7f94 18165754

[21] Pfirrmann CW, Metzdorf A, Zanetti M, Hodler J, Boos N. Magnetic resonance classification of lumbar intervertebral disc degeneration. Spine. 2001;26: 1873–1878. 11568697 10.1097/00007632-200109010-00011

[22] Jones AD, Wafai AM, Easterbrook AL. Improvement in low back pain following spinal decompression: observational study of 119 patients. Eur Spine J. 2014;23: 135–141. 10.1007/s00586-013-2964-5 23963487 PMC3897808

[23] Solberg T, Johnsen LG, Nygaard ØP, Grotle M. Can we define success criteria for lumbar disc surgery?: estimates for a substantial amount of improvement in core outcome measures. Acta Orthop. 2013;84: 196–201. 10.3109/17453674.2013.786634 23506164 PMC3639342

[24] Nachemson AL. Disc pressure measurements. Spine. 1981;6: 93–97. 7209680 10.1097/00007632-198101000-00020

[25] Sato K, Kikuchi S, Yonezawa T. In vivo intradiscal pressure measurement in healthy individuals and in patients with ongoing back problems. Spine. 1999;24: 2468–2474. 10626309 10.1097/00007632-199912010-00008

[26] Nachemson A. The load on lumbar disks in different positions of the body. Clin Orthop Relat Res. 1966;45: 107–122. 5937361

[27] Nachemson A, Elfström G. Intravital dynamic pressure measurements in lumbar discs. A study of common movements, maneuvers and exercises. Scand J Rehabil Med Suppl. 1970;1: 1–40. 4257209

[28] Wilke HJ, Neef P, Caimi M, Hoogland T, Claes LE. New in vivo measurements of pressures in the intervertebral disc in daily life. Spine. 1999;24: 755–762. 10222525 10.1097/00007632-199904150-00005

[29] Adams MA, Hutton WC. The effect of posture on the fluid content of lumbar intervertebral discs. Spine. 1983;8: 665–671. 6685921 10.1097/00007632-198309000-00013

[30] McMillan DW, Garbutt G, Adams MA. Effect of sustained loading on the water content of intervertebral discs: implications for disc metabolism. Ann Rheum Dis. 1996;55: 880–887. 9014581 10.1136/ard.55.12.880 PMC1010338

